# Dropping Counter: A Detection Algorithm for Identifying Odour-Evoked Responses from Noisy Electroantennograms Measured by a Flying Robot

**DOI:** 10.3390/s19204574

**Published:** 2019-10-21

**Authors:** Bluest Lan, Ryohei Kanzaki, Noriyasu Ando

**Affiliations:** 1 Research Center for Advanced Science and Technology, The University of Tokyo, 4-6-1 Meguro-ku, Komaba, Tokyo 153-8904, Japankanzaki@rcast.u-tokyo.ac.jp (R.K.); 2 Department of Systems Life Engineering, Faculty of Engineering, Maebashi Institute of Technology, 460-1 Kamisadori-cho, Maebashi, Gunma 371-0816, Japan

**Keywords:** odour, robot, electroantennogram, signal processing, insect, drone

## Abstract

The electroantennogram (EAG) is a technique used for measuring electrical signals from the antenna of an insect. Its rapid response time, quick recovery speed, and high sensitivity make it suitable for odour-tracking tasks employing mobile robots. However, its application to flying robots has not been extensively studied owing to the electrical and mechanical noises generated. In this study, we investigated the characteristics of the EAG mounted on a tethered flying quadcopter and developed a special counter-based algorithm for detecting the odour-generated responses. As the EAG response is negative, the algorithm creates a window and compares the values inside it. Once a value is smaller than the first one, the counter will increase by one and finally turns the whole signal into a clearer odour stimulated result. By experimental evaluation, the new algorithm gives a higher cross-correlation coefficient when compared with the fixed-threshold method. The result shows that the accuracy of this novel algorithm for recognising odour-evoked EAG signals from noise exceeds that of the traditional method; furthermore, the use of insect antennae as odour sensors for flying robots is demonstrated to be feasible.

## 1. Introduction

Odour tracking is an important ability for living creatures as they can use the olfactory information for finding foods and mates [1]. For a mobile robot, this sort of technique can also be applied and used in various circumstances. For example, a robot that detects carbon dioxide can determine the origin of a fire and extinguish it. Given that carbon dioxide is also a product of breathing, the robot can be employed to find earthquake victims underground. Drug-sniffing robots can be used in airports instead of spending time grooming sniffer dogs. Such robots have not yet been realised; however, insects can perform these tasks. Carbon oxide and skin volatiles are important cues for mosquitos to find human hosts [2]. A type of jewel beetle can detect the smell of smoke and find a forest fire to lay its eggs [3]. Furthermore, the odour learning ability of honeybees can be used for detecting illicit drugs [4]. In addition to their high sensitivity to specific odours, most of the insect species can fly and find an odour source from a distance [5]. Therefore, the odour sensing and tracking abilities of flying insects are part of the goals of robotic odour searching. In robotics, an unmanned aerial vehicle is expected to overcome the limitation of terrain and complete tasks within a shorter time than that required by land robots if it has the capability to sense odour [6,7]. However, the slow reaction speed of gas sensors is a big problem either on land or flying robots, which restrains the control speed of the robotic system.

As odourants are intermittently distributed in the air, fast response and recovery time of sensors are important for tracking patchy distributions [8]. However, in particular, the recovery time of conventional gas sensors, such as semiconductor and conductive polymer sensors, is not sufficiently fast to resolve temporary changing odour information [9,10] compared to insect antennae (i.e., the olfactory organs of insects). This makes the sensing process more difficult. Although odour detection strategies for flying insects have been extensively studied [11,12,13], it is difficult to implement their algorithms to robotic platforms owing to the use of artificial sensors [6]. In contrast, using the electroantennogram (EAG) technique for measuring the nerve signal output of an insect’s antenna for a given odour [14] is a direct method that employs an insect’s ability to capture olfactory information at a high speed and rapid recovery rate.

The EAG has been used as a biosensing method owing to its high sensitivity [15]. It typically involves inserting signal and reference electrodes to the proximal and distal ends of the antenna, respectively, and then a negative potential (from several hundred microvolts to a few millivolts based on the concentration) is detected in response to an odour stimulus [16]. The advantage of using an insect antenna for robotic odour detection is its high temporal resolution owing to its short recovery time, along with its high sensitivity [17]. To overcome the slow recovery time of the semiconductor sensors, an algorithm using a system modelling approach of the sensor [18] or the derivative of the sensor output to decompose odour bouts has been developed [19] and implemented in small-sized drones for odour searching tasks [20,21]. Recent studies reported that some semiconductor gas sensors can resolve 2–5 Hz of odour pulses [21,22]; however, these values are still lower than the temporal resolution of insect antennae (more than 100 Hz [23]). The problem of using the EAG for robotic odour searching is that the measurement is strongly influenced by electrical and mechanical noises caused by robot movement, and the attenuation of odour signals because the antennal tissue dries and dies if the antenna is isolated. Therefore, experimental procedures to improve the stability and longevity of the EAG measurements and signal processing to discriminate odour signals from noisy signals have to be developed.

Kuwana et al. [24,25] showed the possibility of realising odour tracking with isolated silkmoth (*Bombyx mori*) antennae mounted on a mobile robot. They reported that the baseline drift (low frequency noise) presumably due to antenna drying and high-frequency noise caused by robot motors interfered with signal detection [26]. To reduce these noises, a drift canceler and a bandpass filter were implemented in the amplifier circuit. They also reported that the bandpass filter could not separate the EAG signal from the baseline drift because their frequency components were sometimes the same. Instead, they adjusted the diameters of silver wire electrodes to tightly fit on the diameters of both ends of the antenna to prevent them from drying, which resulted in the reduction of the drift. These improvements were necessary to drive the reactive searching algorithms based on silkmoths because odour detection was defined based on a fixed threshold by a comparator. Martinez et al. [17,27] improved the longevity of the EAG measurement on a mobile robot. They recorded EAG not from an isolated moth antenna but from an intact one (whole-body preparation) with a glass electrode.

Although EAG measurements on a drone have not yet been conducted, the improvements in these experimental procedures cannot simply be applied to flying robots. This is because the mechanical noise caused by the self-generated wind from the propellers and its own three-dimensional manoeuvres prevent stable EAG measurements from being obtained using sharp electrodes. The electrical noise caused by multiple motors and onboard circuits also interferes with the measurement results. Furthermore, as the payload of a drone is limited, the size and weight of the apparatus for the EAG measurement must be as light as possible and the computational load for signal processing must also be minimised to save batteries. These requirements imply that both the measurement device and the algorithms for signal processing must be as simple as possible. In this study, to meet the requirements, we measured EAGs on a drone and investigated the characteristics of signals using a custom-built antenna holder. Based on the experiment, we then developed simple algorithms to eliminate noise and recognise the odour-evoked EAG signal from noise.

## 2. Electroantennogram Measurement

### 2.1. Hardware

#### 2.1.1. Biosensor

**Figure 1:**
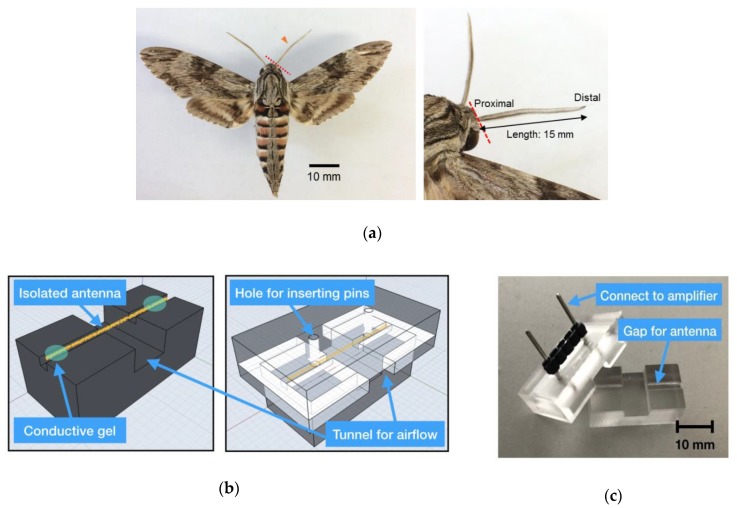
Hawkmoth antenna and antenna holder setup. (**a**) The antennae of the male hawkmoth, *A. convolvuli*. The antenna was cut at the base (red dashed line; the antenna is indicated by an arrowhead). The total length of the antenna was around 15 mm and its maximum diameter was about 1 mm. (**b**) Drawings of the antennal holder. The isolated antenna was placed on the lower part, electroconductive gel was added on both ends (left panel), the upper part covered the lower one (right panel), and then pins were inserted through two holes. The pins attached the proximal and distal ends of the antenna. (**c**) Appearance of the antennal holder.

In the EAG measurement experiments, 3- to 10-day old adult male hawk moths (*Agrius convolvuli*) were used. Either side of the antenna was cut at the basal segment with a pair of fine scissors and used as a biosensor (Figure 1a). The sensor should be designed for easy use and fast setup to avoid the cell from dying. Instead of inserting metal or glass electrodes inside the antennal tips with conventional techniques [16], we made a 24 × 14 × 11.5 mm customised acrylic glass-based antenna holder (Figure 1b,c) to simplify the preparation setup, which only took less than 1 min. The lower part was used for placing an isolated antenna, and the upper part was used as a lid to ensure that the antenna inside was fixed. Furthermore, on the top of the lid, there were two pins, which acted as electrodes and enabled us to connect the antenna to a preamplifier circuit directly.

After putting the isolated antenna in the middle gap of the holder, electrically conductive gels (Spectra 360, Parker Laboratories, NJ, USA) were simply attached to two tips and the lid was closed; then, the preparation was finished. Moreover, when the lid was closed, there was merely a small tunnel for the airflow, which could decrease the influence of the surrounding unstable airflow. This holder could tightly fix the antenna’s position to reduce extrinsic mechanical noise and facilitated the biosensor setup.

**Figure 2:**
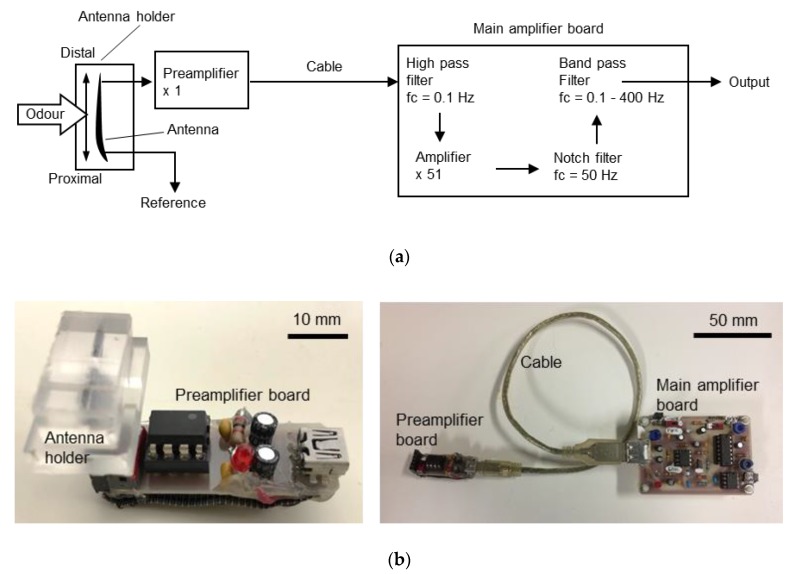
Custom-built electroantennogram (EAG) amplifier. (**a**) Schematic of the amplifier. (**b**) The antenna holder attached to the preamplifier board (left) and amplifier boards (right).

To amplify and filter the EAG signals, we used custom-build amplifier boards consisting of a preamplifier and a main amplifier (Figure 2a). The preamplifier was a voltage-follower with a unity gain, and the main amplifier had a voltage gain of 51 V/V. To eliminate 50 Hz noise from the alternate current, a notch filter was implemented. High- (cut-off: 0.1 Hz) and low-pass (cut-off: 400 Hz) filters were also implemented to remove the direct current (DC) offset and high-frequency noise. After setting up the antennae, the antenna holder was directly connected to the preamplifier board with pins (Figure 2b).

#### 2.1.2. Flying Robot

We used a 520 (L) × 520 (W) × 127 (H) mm drone (AR.Drone 2.0, Parrot, France) for the study considering the payload and programmability [28]. The drone is able to carry up to 200 g objects, which is enough for mounting the sensors and electric circuit boards on it. As the final goal of the study is to make an odour-tracking robot, the hardware of the robot was also under our consideration. With an ARM Cortex A8 1 GHz processor and a DDR2 1 GB memory, the drone can follow the commands that we send via wi-fi without difficulty. Still, the built-in accelerometer (3 axles, accuracy of ±50 mg) and a gyroscope (3 axles, accuracy of 2000°/s) enable us to stabilise the hovering drone at the same position by building a proportional-integral-derivative controller (PID controller).

### 2.2. Experiment

#### 2.2.1. Experimental Setup

**Figure 3:**
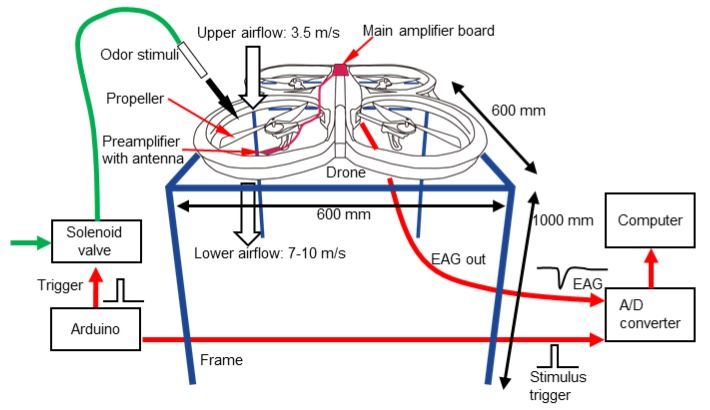
Tethered flight setup illustration. The preamplifier with the antenna was fixed under the propeller.

This study focuses on the noisy EAG signal recorded from the flying drone. However, even if we fixed the position of the odour source, because a flying drone would not stay at a certain position, conditions such as the distance between the odour source and the drone would change every second. Tethered flight experiments can reduce the uncertain factors, simulate the flying condition, and make us concentrate on signal detection. To simplify, in the experiment, we fixed the drone on a set of frames sized 600 (L) × 600 (W) × 1000 (H) mm (Figure 3). We aimed at collecting EAGs from the antenna under the hovering airflow condition. When the propellers are not active, it can be considered that the drone is on the ground. However, once the propellers start to rotate, we can simulate the condition of a flying drone. The altitude of 1000 mm is the normal one when the drone is autonomously hovering. To verify the result of the free hovering flight [29], we measured the wind speed over a propeller and under it during a tethered flight using a hot-wire anemometer (DT-8880, CEM, Shenzhen, China). The result just matched the theory in that the later reading value was around 7–10 m/s, which was almost double the former (Figure 3), as the sensor can catch the chemical plume more than twice and enhance the sensing opportunities. Therefore, we fixed the preamplifier board under the propeller (front-right) so as to capture the odourant with the antenna. We also confirmed that the position of the antenna can catch an odour coming through the propeller using a photoionisation detector (miniPID 200B, Aurora Scientific, Aurora, ON, Canada). The main amplifier board was attached to the upper side of the body. The total weight of the amplifier board including a battery was 54.4 g, which was sufficiently light for the drone to carry if it flies freely.

For measuring the EAG response, we used the major component of the homogeneous female sex pheromone of hawk moth [(E, E)-11,13-hexadecadienal, Shin-etsu Chemical, Tokyo, Japan] [30] as a testing odourant. The treatment of the odourant and the olfactometer were based on the previous work of EAG measurements in silkmoths [31]. The odourant was dropped on a piece of filter paper (10 × 20 mm) and inserted into a glass tube (φ5 mm). The amount of the odourant per pipette was 200 µg. To release the odourant above the front-right propeller of the drone, a glass tube containing the odourants was attached to a micromanipulator and was fixed on the frame. The position of the tube was then adjusted so as to obtain odour responses with large amplitudes repeatedly. 

#### 2.2.2. Experimental Condition

We released the odourant at a constant frequency in a single experiment (200 ms of the odourant puffing at different frequencies, 0.25, 0.5, 1 Hz; flow rate, 1 L/min) using a solenoid valve (VDW350-6G-2-01, SMC, Tokyo, Japan) controlled by a microcontroller board (Arduino Uno, Arduino, Italy). The output signal from the amplifier was acquired by an analogue to digital (A/D) converter (PowerLab SP8, ADInstruments, Dunedin, New Zealand) at a sampling rate of 10 kHz for recording all the details (Figure 3). The trigger signals driving the solenoid valve, regarded as input signals, were also input to the A/D converter. We acquired EAGs from 10 moths in total.

## 3. Signal Processing

### 3.1. Characteristics of Signals

The requirements of signal processing for drone odour tracking is that the computational load must be as minimised as possible. As the payload of the drone is limited, the low load enables us to select small-sized but not so powerful microcontroller boards. The low computational load also leads to low power consumption and can save battery life. Furthermore, a quick response to the change in odour distribution is necessary for tracking an odour plume, and signal processing with a sufficient time resolution and short time delay are also required. Therefore, we aimed at designing a simple algorithm for signal processing, with which we can robustly detect an odour response from the EAG recording even with the electrical and mechanical disturbances of the flight.

Before we start to build algorithms, we need to understand the characteristics of the EAG for developing a suitable one. Furthermore, picking up a reasonable sample rate is also important for minimising the load of the microcomputer as it also influences the time delay. To recognise if the sensor captures odourants or not, a simple method is to set a threshold. Ideally, once the signal reading value reaches the assigned threshold, it can be considered that the antenna was stimulated by odourants. However, even though the antennae of insects react to specific odours only, the EAG recordings were still influenced by extrinsic noises such as the electrical noise associated with the alternating current or the drone itself and the mechanical noise produced by the propellers. Basically, there are two methods that can increase the signal-to-noise ratio (SNR) of the odour-evoked EAG response: enhancing the EAG signal and reducing noise. Previous works [32,33] showed that the use of multiple antennae in either series or parallel increases the signal amplitude or the signal-to-noise ratio of odour responses. This method would make it easier to discriminate odour responses from other signals, including extrinsic noises. However, preparing multiple antennae on an electrode requires time for setup and the individual differences among multiple antennae would influence the improvement of the SNR. In this study, instead of using a biological method to enhance the SNR, we tried to build a computational algorithm for detecting an odour response from the EAGs.

A series of experiments were carried out for determining methods that can solve the noise-influence problem. To know the frequency component of the different situations, we conducted measurements with the following four conditions:

Type 1: Propellers off, no pheromone (*noise_off_*)
Type 2: Propellers off + pheromone stimulus (*noise_off_* + *sig_odour_*)
Type 3: Propellers on, no pheromone (*noise_on_*)
Type 4: Propellers on + pheromone stimulus (*noise_on_* + *sig_odour_*)

**Figure 4:**
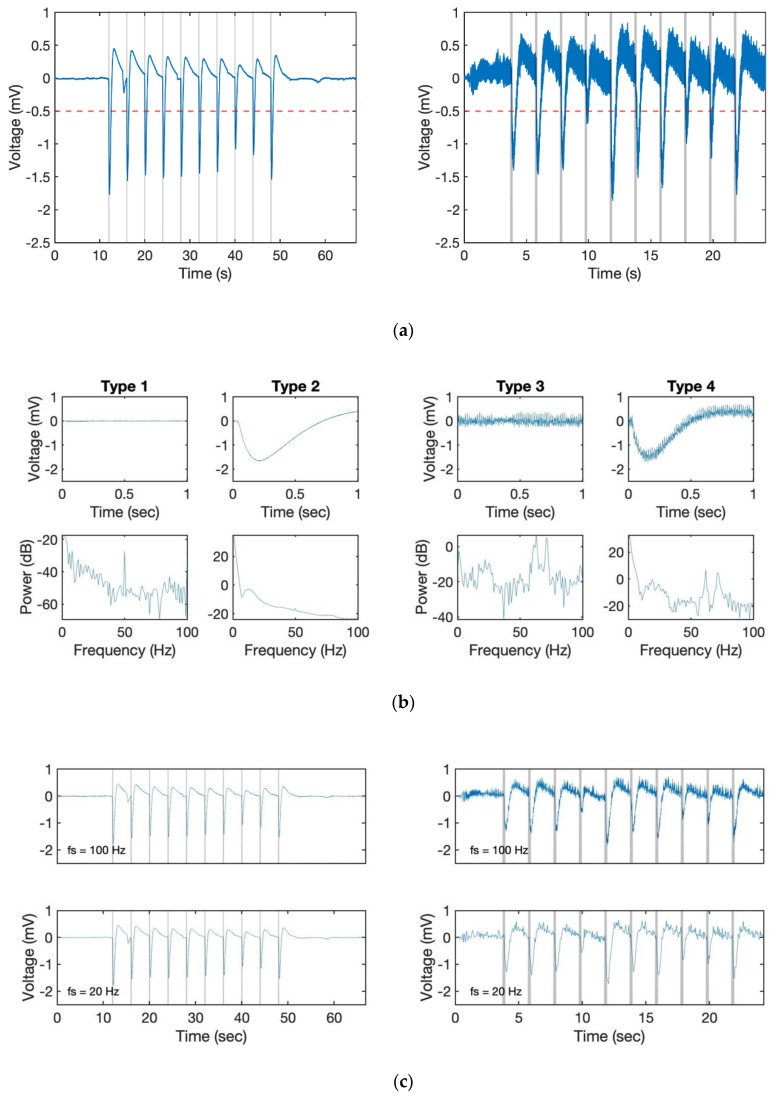
Characteristics of EAG signals recorded on the drone. (**a**) EAG signal when propellers were off (Type 2, left) and on (Type 4, right) with 10 kHz sample rate. The red dashed line is a fixed threshold at −0.5 mV and the grey area represents the odour stimulation (0.25 Hz and 0.5 Hz). (**b**) A typical single odour response (upper row, the odour was applied only in Type 2 and 4) and power spectral density (PSD, lower row) of different types of signal in a second. (**c**) The signal of (**a**) resampled at 100 Hz (upper row) and 20 Hz (lower row) for comparison.

Generally, without a heavy disturbance, the EAG response on the drone can be considered as a combination of these four basic signals (see Figure 4a,b). Based on the results, Figure 4b shows the power spectral density (PSD) analysis of these four types of signal recorded at a 10 kHz sampling rate. From the result, we found out that the strongest signal appeared close to 0 in all cases. It is worth mentioning that a signal around 50–70 Hz appeared in all 4 cases due to the power line hum. Even though we had already used a notch filter to avoid this phenomenon, it could not be erased entirely. However, as the major component of the odour response signal was located at less than 10 Hz (see type 2 and 4 compared to type 1 and 3 in Figure 4c), we did not need to set the sampling rate so high. Figure 4c shows the result with 100 and 20 Hz. By comparing it with the result in Figure 4a, we can still identify each response even with a lower sampling rate. Furthermore, the signal became clearer partly because the high-frequency noise disappeared without using extra filters. At the same time, as the number of sampling points was reduced, the load of the microprocessor decreased, which also improved the performance. However, the lower sampling rate led to a subsequent reduction in the lower signal processing and control frequency. As the following algorithms still need additional data for computation, we therefore set the control frequency of 100 Hz in this study.

### 3.2. Filter

**Figure 5:**
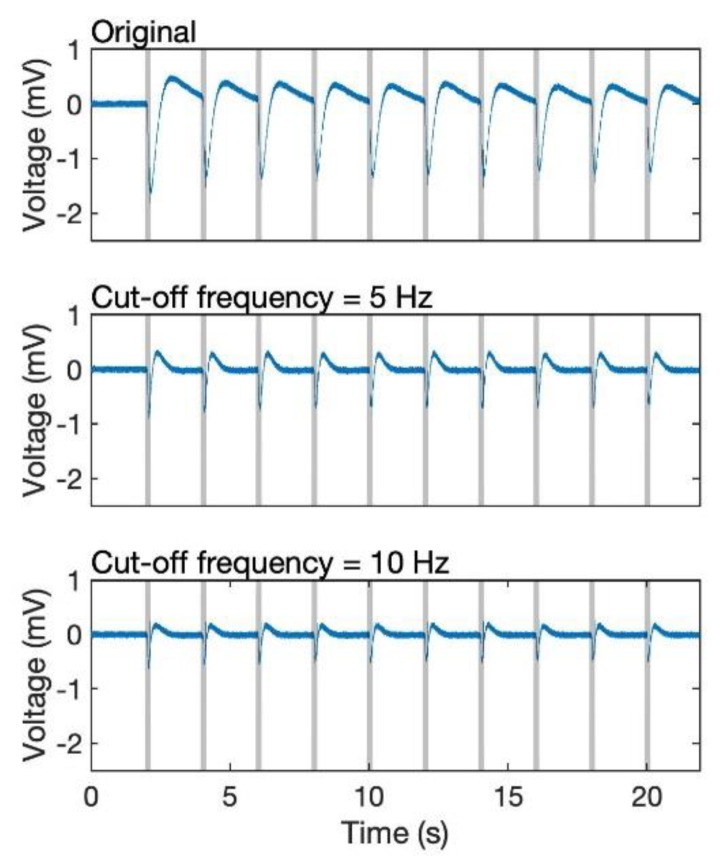
EAG signal passed through a high-pass filter. The original signal (type 2) sampled at 100 Hz (top), filtered at a cut-off frequency of 10 Hz (middle) and 5 Hz (bottom).

Because odourants are distributed intermittently in the air, the odour response of the EAG is a pulsed signal. The frequency of odour contact depends on the distance from the odour source and strongly influences the tracking trajectories of insects [34]. Therefore, detecting the odour pulses from the EAG is the goal of signal processing. The most simplified method for detecting an odour stimulated signal would be to set a fixed threshold. Once the sensor reading reaches the assigned value, it would be considered as a gaseous signal. From Figure 4a, the amplitude of a single response is on the interval [−1.8 mV, 0.5 mV]; however, a fixed threshold (for example, −0.5 mV) for verifying whether the biosensor detects odourants or not leads to an erroneous result owing to the bias of the EAG signal. This is because the frequency component of EAG response is less than 10 Hz (Figure 4), it is overlapped with that of the bias, which was also reported by Kuwana et al. [26]. Therefore, even though a high pass filter can be used for removing the DC component, it also decreases the amplitude of the EAG responses as their major frequency is extremely low (Figure 5). Thus, we build an additional algorithm that can robustly achieve odour detection even if the bias does not go back to the original, and it should have the ability for us to find out the timing of the stimulus.

**Figure 6:**
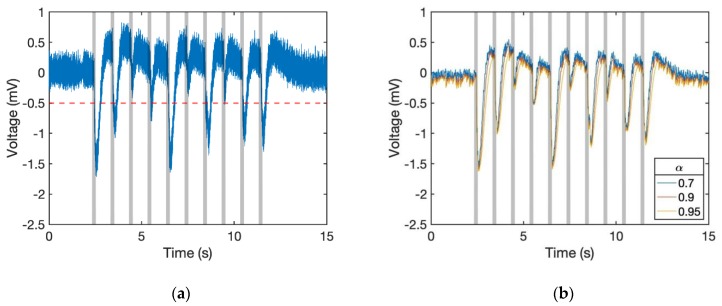
Application of the conditional exponential moving average (EMA) filter. (**a**) Raw data sampled at 100 Hz. (**b**) Filtered data. The conditional EMA filter reduced the high-frequency noise, whereas negative peaks of odour response remained. The results of different *α* are shown.

Figure 6a shows the situation with drifting standard voltage. As the signal around the 5th second did not remain at 0 mV, once we set the threshold at −0.5 mV the response cannot be detected. To solve the problem, we made the positive response smaller than the negative one by using a computation method. Because the EAG response is negative, we can apply an exponential moving average (EMA) filter, which is also known as a type of infinite impulse response (IIR) filter, onto the system for making the signal smoother. At time period *n*, the filtered data *y* can be known by the original data value *x* through Equation (1).

*y*[0] = *x*[0] *y*[*n*] = *αx*[*n*] + (1 − *α*)*y*[*n* − 1], *n* > 0 (1)

where *α* is a smoothing factor, and 0 *< α <* 1.

Afterwards, to make the rising and dropping response more different, we introduced the conditional EMA filter into the system. The smoothing factor *α* of the dropping signal should be set larger than the rising one. To simplify, we assigned the smoothing factor as 1 − *α* for positive responses as shown in Algorithm 1. By using this algorithm, the signal became smoother and the negative parts were enhanced. Figure 6b shows the dataset of 6a that passed through this filter. The rising signal became weaker than the others and some of the fluctuating parts even disappeared.

**Algorithm 1.** Conditional EMA filter*sig*[*n*] ← input**if***sig*[*n*] − *sig*[*n* − 1] *<* 0 **then**  *sig*′[*n*] ← *α sig*[*n*] + (1 − *α*) *sig′*[*n* − 1]
**else**
  *sig*′[*n*] ← (1 − *α*) *sig*[*n*] + *α sig′*[*n* − 1]
**end if**
output ← *sig*′[*n*]

### 3.3. Detecting Algorithm

Given that a fixed threshold does not effectively work in this case, we built the additional algorithm named dropping counter (D-Counter) for detecting the odour-evoked response (Algorithm 2). As the response signal appears as a negative potential, the basic idea of the algorithm is to count how many points are dropping below a reference value and use this as a flag to determine if it was the signal that we want. To limit the counting time, a window was created during the process. In the window, the first value (*sig*″[0]) was assigned as the reference point. This value would be compared with all the other points (*sig*″[*i*]) and the number of values (*d_counter_*) that are smaller than itself are found out. The overall steps can be concluded in Algorithm 2.

**Algorithm 2.** D-Counter*sig*″ ← *sig*′ inside the window*d_counter_* ← 0∆*_max_* ← 0**for** each *i* in *sig*″ **do**  ∆ ← *sig*″[*i*] − *sig*″[0]  **if** ∆ *<* 0 **then**   *d_counter_* ← *d_counter_* + 1   **if** ∆ *<* ∆*_max_*
**then**    ∆*_max_* ← ∆   **end if**  **end if**
**end for**
**if** ∆*_max_ > th*
**then**  *d_counter_* ← 0
**end if**
output ← *d_counter_*


In the algorithms above, there are several variables that we can adjust: sampling frequency *f_s_*, smoothing factor *α*, size of window *s_win_*, and threshold *th*. By assigning different values to these parameters, we can control the sensitivity of the sensor and find out the timing of the stimulus.

## 4. Results

**Figure 7:**
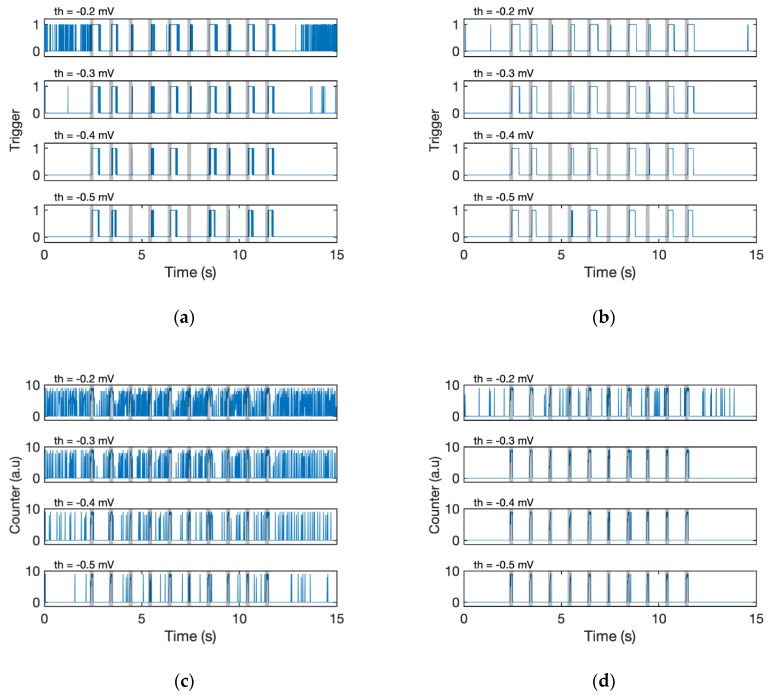

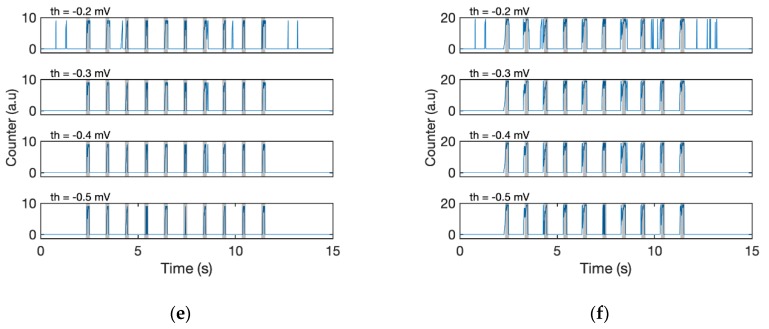
Results of different algorithms. The blue line indicates output of each algorithm. The original data are shown in Figure 6. (**a**) Output of the original signal with the fixed threshold method (Algorithm 3, the output is binary, 0 or 1). The results of different thresholds (*th* = [−0.5 mV, −0.2 mV]) are shown. Note that *th* in the fixed threshold is an absolute voltage, whereas *th* in D-counter is a relative one (see Algorithm 2 and 3) (**b**) EMA filtered signal (α = 0.7) with the fixed threshold. (**c**) Original signal with D-Counter (*s_win_* = 0.1 s). (**d**) EMA filtered signal (*α* = 0.7) with D-Counter (*s_win_* = 0.1 s). (**e**) EMA filtered signal (*α* = 0.9) with D-Counter (*s_win_* = 0.1 s). (**f**) EMA-filtered signal (*α* = 0.9) with D-Counter (*s_win_* = 0.2 s).

To evaluate the effectiveness of the proposed algorithms, the detection results obtained with different combinations of the filtering (with/without EMA filter) and detection algorithm (Fixed threshold or D-counter) were compared (Figure 7; for fixed threshold, see Algorithm 3). The simple fixed threshold could not recognise all the stimuli, whether the signal passed through the EMA or not (Figure 7a,b). Even if we specified a lower threshold, the algorithm failed to detect odour responses with small amplitudes (third and sixth responses, see the original waveform in Figure 6), and it was still challenging for us to detect each stimulus. The result of a stand-alone D-Counter that did not contain any EMA filters was also tested; however, the calculation result is too noisy for us to recognise which is the original timing of stimuli (Figure 7c). Meanwhile, the D-Counter with EMA filters adequately detected all 10 responses regardless of the different window sizes (*s_win_* = 0.1 or 0.2 s) and smoothing factors (*th* = [−0.5 mV, −0.3 mV], Figure 7d–f).

To find out the performance of each algorithm and the influence of the parameters, we examined the similarity between the initial stimulus signal (i.e., odour reception) and these results by computing their cross-correlation coefficients. As the detection result and stimulus signal are not synchronised, we compared the maximum coefficients. We corrected data from 10 individual moths with different stimulus conditions [stimulus frequency: 0.25 Hz (*N* = 4), 0.5 Hz (*N* = 2), 1 Hz (*N* = 4); stimulus duration: 200 ms, number of stimuli: 10, *f_s_* = 100 Hz].

**Figure 8:**
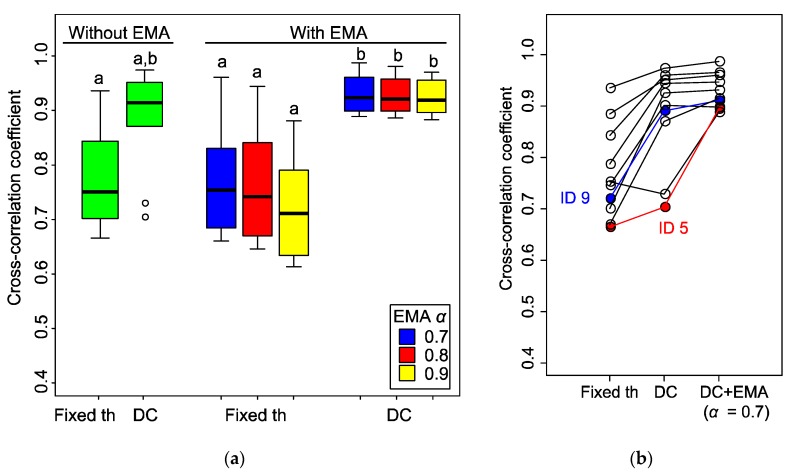
Cross-correlation between different algorithms (*N* = 10). The maximum coefficients when considering the time-shift, where *th* = −0.5 mV and *s_win_* = 0.2 s, are shown. (**a**) Box plots of cross-correlation coefficients; the top and bottom sides of a box indicate the first and third quartiles, and the bar represents the median. The whiskers indicate the maximum or minimum values. Plots with different letters indicate significant differences between them (*P* < 0.05, Steel–Dwass test). Fixed th: fixed threshold method (Algorithm 3), DC: D-Counter (Algorithm 2), EMA: conditional EMA filter (Algorithm 1). (**b**) Individual difference of cross-correlation coefficients by different algorithms. The plots connected with lines indicate each moth. The original waveforms of EAG signals of ID 5 (red) is shown in Figure 6 and ID 9 (blue) is shown in Figure 4a, right panel.

The results (Figure 8a) indicated that the application of the D-Counter to both the original and EMA filtered signals increased the cross-correlation coefficients. With EMA filter, there were significant differences between the coefficients in the fixed threshold and D-Counter methods (*P* < 0.05, Steel–Dwass test), whereas there was no significant difference between them without the EMA filter, presumably owing to the large individual differences (*P* = 0.23). The application of the EMA filter did not influence the median of the coefficients (*P* > 0.05 for both the fixed threshold and D-Counter), while it reduced the range of data distribution if it was utilised with the D-Counter (see two outliers in D-Counter without EMA). Although there were no significant differences in coefficients between the different smoothing factors *α*, the smaller *α* (0.7) slightly increased the median of the coefficients.

The large individual difference observed in the D-Counter without the EMA filter was due to the characteristics of each EAG signal. In Figure 8b, the antenna from the moth ID 9 showed a high coefficient value (0.72 to 0.89) after applying the D-Counter only, whereas the increase was small in ID 5 (0.67 to 0.70) and that of the other sample decreased. The EAG signal from ID 9 showed relatively constant amplitudes of odour responses and the baseline was stable (original waveform, see Figure 4a, right panel), whereas the response amplitudes and baseline were unstable in ID 5 (Figure 6). Although there were individual differences in the original EAG signals, the results indicated that the D-Counter with EMA filter could reduce the differences and estimate the timing of odour reception with high similarity to the input stimuli.

**Algorithm 3.** Fixed thresholdsig[n] ← input**if***sig*[*n*] < *th*  *trig*[*n*] ← 1
**else**
  *trig*[*n*] ← 0
**end if**
output ← *trig*[*n*] 

## 5. Discussion

In this study, we developed a simple EAG detection algorithm for flying robots. The conditional EMA filter and D-Counter provide a unique algorithm that can be adopted for handling different cases or changing the sensitivity by adjusting the smoothing factor *α*, window size *s_win_*, and threshold *th*. Compared with the simple fixed threshold detection, the D-Counter conferred a high accuracy of detection (quantified using a cross-correlation coefficient between the detection output and stimulus input signals). Moreover, applying the conditional EMA filter with an appropriate smoothing factor *α* can reduce the individual differences even if it is applied to signals with variable signal amplitudes and the baseline. The individual diversity in bio-sensing technology has been the major problem as each individual has different characteristics. Such diversity is caused not only by the native difference of individual moths but also by the differences among antenna preparations. Therefore, the reduction in response diversity is also the advantage of our algorithm. In addition to the development of signal processing, our simplified and easy-to-set up experimental technique using an antenna holder can unify the conditions of antenna preparations and reduce the individual differences. How to minimise the effect of the individual differences is the great challenge when utilising insect antennae for practical use as chemical sensors. Improvements in both signal processing and experimental procedure are required.

The odour-evoked EAG response encodes multiple information of an odour filament. The fast response onset indicates the timing of odour arrival, the amplitude indicates odour concentration, the duration of a negative potential indicates the duration of odour contact, and the waveform of a single response can be altered by different odourants [14]. However, all the information would be available only when an odour is applied under a well-controlled condition and the EAG is obtained using the same preparation. In particular, unlike gas sensors, it is difficult to estimate the absolute odour concentration from the amplitude of EAGs because the amplitude of an EAG to a certain odour concentration varies by individual preparations and their conditions, and the signal decays even if the antenna is in a continuous odour flow. Although our algorithm only tells us the onset and offset of each odour response, this will not be a problem for odour tracking. Because the duration of a single odour contact for flying insects is short (several hundred milliseconds or less), the detection of the odour onset and offset would be more reliable than odour concentration [12,13]. Therefore, as long as we use odour-searching algorithms of flying insects in which the behaviours are triggered by odour onset or offset, our algorithm of signal processing will be effective. However, our algorithm cannot be used for a searching algorithm based on the concentration gradient of odour, such as osmotropotaxis, in which an insect turns to the side of higher odour concentration (i.e., the side of an odour source if the odour flow is continuous) [35,36]. However, whether flying insects during manoeuvres avail the odour gradient during such a short period is still in question, even though they are capable of detecting it [37].

Not only the odour concentration, but the timing of response onset also tells us the side of an odour source if bilateral information is acquired by two organs spatially separated. It has been reported that sharks and silkmoths turn based on the time difference of odour arrivals between their left and right olfactory organs [31,38]. Because the timing of response onset of the EAG and its temporal dynamics are highly reproducible [39], the time difference in EAG onsets between two antennae spatially separated can be used to determine the side to turn. Kanazaki et al. [40] implemented the time difference of EAG responses between two antennae to the network model on an antennae-loaded land robot. Furthermore, whether the timing of different odour arrivals is synchronised or not is supposed to be a cue for insects to recognise the odourants as mixed or not [39,41]. Therefore, the timing of odour onset is solely meaningful, and how to utilise it for efficient searching should be investigated in the future.

The time necessary for processing signals in our algorithm is mainly constrained by the window size *s_win_*, whereas the computational load for the algorithm itself is low. We showed that either *s_win_* = 0.1 s or 0.2 s can output an accurate estimation of the olfactory input, which correspond to 0.1 s or 0.2 s of latency if the algorithm is operated in real time. Although the time delay should be as minimised as possible, it is worth noting that the response delay of a surge (the upwind flight triggered by an odour contact) is approximately 0.2 s in moths and flies [12,13]. The allowance of response delay for successful odour source localisation was estimated to be 0.4 s, which was investigated employing a silkmoth-driven land robot [42]. Altogether, considering the time required for sensory-motor control of insects, the time delay for signal processing in our algorithm does not prevent us from availing odour-searching algorithms of insects for the drone.

Flying robots such as drones, have better manoeuvring capabilities than land robots, which allows them to complete tasks faster. However, during odour detection, the turbulence they create makes it difficult for them to accomplish their tasks. The additional airflow not only blows the odour away but also influences its distribution. Although an antenna-based biosensor can increase their sensitivity, the EAG signal is easily affected by the noise as well. The algorithm proposed in the paper minimises the disturbance from the airflow and enables us to recognise the timing of stimulation. Nevertheless, the development of tracking algorithms to overcome other negative impacts will be considered in future studies.

This study raised a new odour localisation method with a flying robot employing the insect antenna as a biosensor. As the EAG technique provides another approach for capturing an odour plume, it has great potential in the future. Although it is challenging for a single antenna to differentiate between specific odorants as it senses multiple odorants, odour discrimination based on multivariate analyses of EAGs using multiple antennae from different species has been reported [43,44]. Furthermore, the recent advancements in genetic tools enables us to alter the type of olfactory receptors on the sensory neurons in moth antennae, which in turn alters the selectivity to a specified odour [45]. This technique will be of a great advantage when using insect antenna to detect other odorants, such as odorants related to hazardous or explosive materials, drugs, fires, and disaster victims.

## 6. Conclusions

This paper introduced a simple counter algorithm for a flying robot to recognise EAG responses. To design the algorithm, we collected and studied the characteristics of EAG signals by analysing their frequency components. Based on the results, we first used a conditional EMA filter to enhance the negative responses by assigning a lower weight to the positive responses. As the EAG responses were negative, the D-Counter algorithm created a window and compared the values inside it. The counter increased by one when a value was smaller than the first one and then reconstructed the odour stimuli. A comparison of our results with those of a traditional fixed threshold method proved that our new design provides a higher cross-correlation coefficient, which also implies that it exhibits a better noise performance.

The series of experiments conducted in this study show that it is feasible to use a flying robot and an insect antenna for odour tracking. With the newly designed algorithms, the noisy EAG signals measured by insect antennae can be converted into valuable data, which can then be utilised for determining the encounter timing of an odour trail. In future work, we will use this information to realise odour localisation.
